# Supplementary material for: Structural and functional analysis of the role of the chaperonin CCT in mTOR complex assembly
Source: Nat Commun. 2019 Jun 28;10:2865. doi: 10.1038/s41467-019-10781-1 (PMC6599039; doi:10.1038/s41467-019-10781-1)
Supplement: Supplementary file 1 — Supplementary Information [file 41467_2019_10781_MOESM1_ESM.pdf]

Supplementary Information

Structural and functional analysis of the role of the chaperonin CCT in mTOR complex assembly

Cuéllar et al.

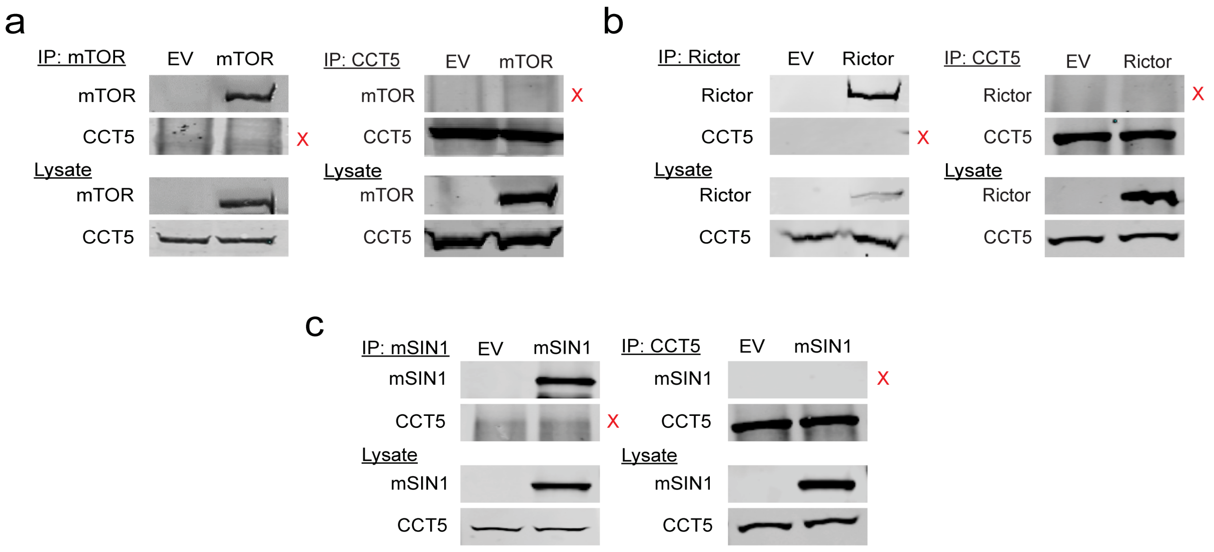

Supplementary Figure 1. mTOR, Rictor and mSIN1 do not bind CCT. Co-immunoprecipitation experiments testing for possible interactions of mTOR **(a)**, Rictor **(b)**, and mSIN1 **(c)** with CCT. The absence of co-immunoprecipitating bands is marked (red X). Representative blots are shown from three separate experiments. Source data are provided in the Source Data file.

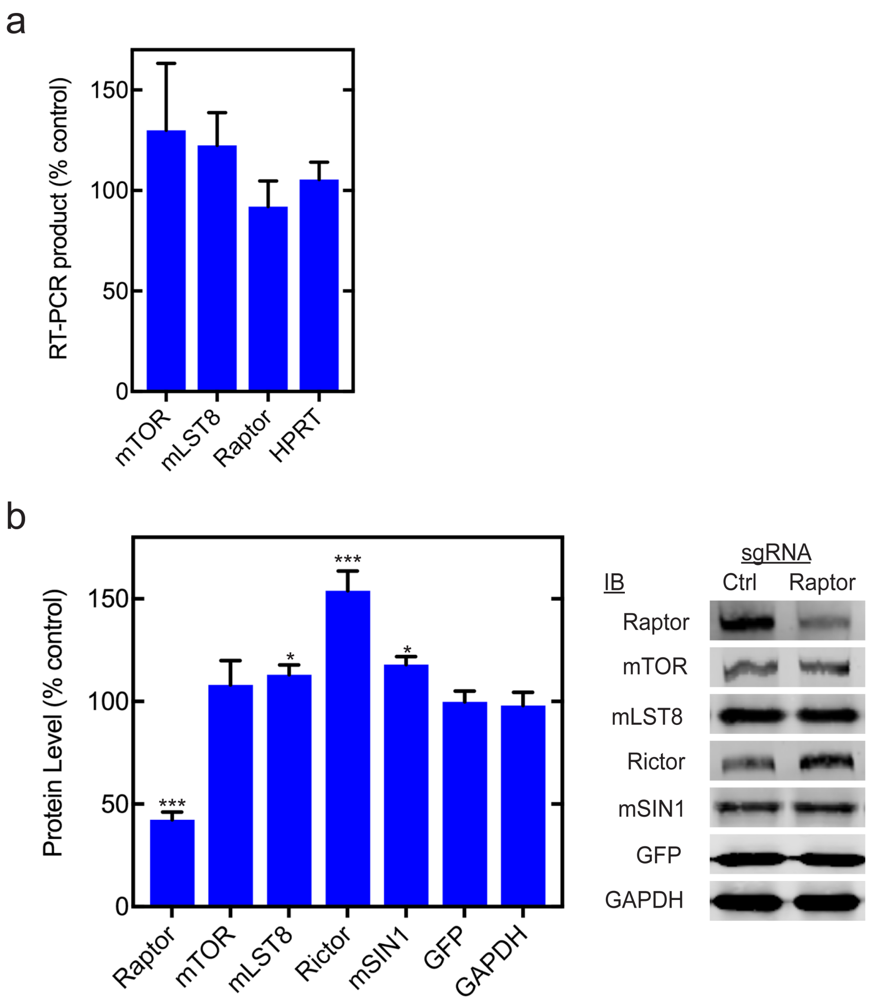

Supplementary Figure 2. Raptor loss increases Rictor expression. **(a)** Effects of CCT loss on mTORC1 subunit mRNA levels. Cells were treated with CCT5 sgRNA or non-targeting sgRNA as in Fig. 2, and endogenous mTOR, Raptor, and mLST8 mRNA levels were measured by qPCR. Data are shown as a percent of the control. **(b)** Effects of Raptor depletion on ectopic expression of mTORC2 subunits. Cells were treated with Raptor sgRNA or non-targeting sgRNA as in Fig. 2 and were transfected with mTORC2 subunits and a GFP control. Lysates were immunoblotted and quantified for expression of mTORC2 subunits and control proteins as indicated. Results are shown as a percent of the control. * $p < 0.05$, *** $p < 0.005$. Source data are provided in the Source Data file.

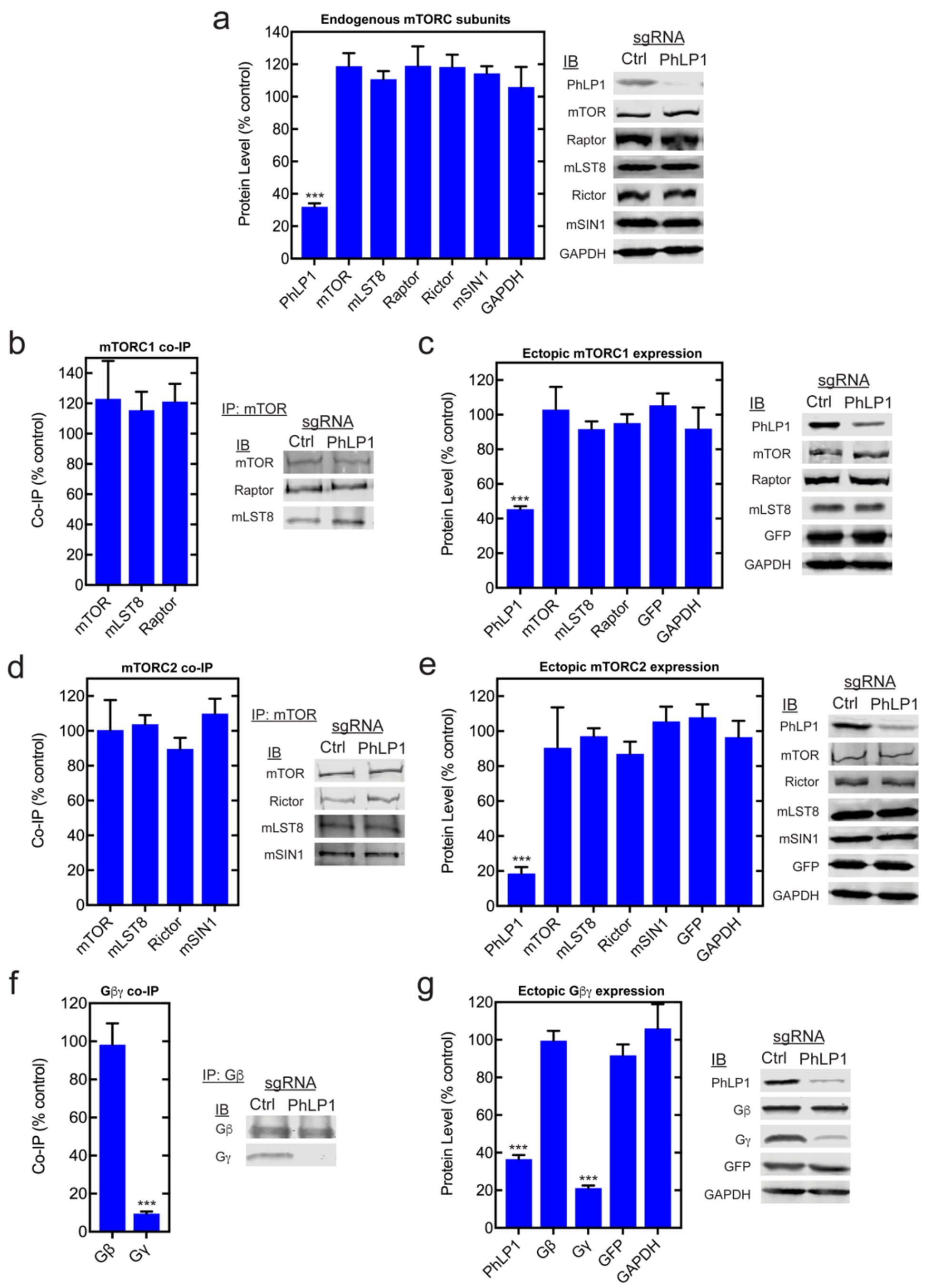

Supplementary Figure 3. PhLP1 does not contribute to mTORC formation. **(a)** Effects of PhLP1 depletion on endogenous mTORC subunit expression. Cells were treated with PhLP1 sgRNA or non-targeting sgRNA and Cas9 as in Fig. 2. Lysates were immunoblotted and band intensities were quantified as indicated. Results are shown as a percent of control cells. Bars represent the average ± standard error. **(b)** Effects of PhLP1 depletion on co-immunoprecipitation of mTORC1 subunits with mTOR. Cells were CRISPR treated and transfected with the indicated mTORC1 subunits and a GFP control. mTOR immunoprecipitates were immunoblotted and quantified as indicated. **(c)** Lysates from the cells in panel b were immunoblotted and quantified for expression of mTORC1 subunits and control proteins as indicated. **(d)** Effects of PhLP1 depletion on co-immunoprecipitation of mTORC2 subunits with mTOR. Cells were CRISPR treated and transfected with the indicated mTORC2 subunits and a GFP control. mTOR immunoprecipitates were immunoblotted and quantified as indicated. **(e)** Lysates from the cells in panel d were immunoblotted and quantified for expression of mTORC2 subunits and control proteins as indicated. **(f)** Effects of PhLP1 depletion on co-immunoprecipitation of Gγ with Gβ. Cells were CRISPR treated and transfected with Gβ, Gγ and a GFP control. Gβ immunoprecipitates were immunoblotted and quantified as indicated. **(g)** Lysates from the cells in panel f were immunoblotted and quantified for expression of Gβ and Gγ and control proteins as indicated. *** $p < 0.005$. Source data are provided in the Source Data file.

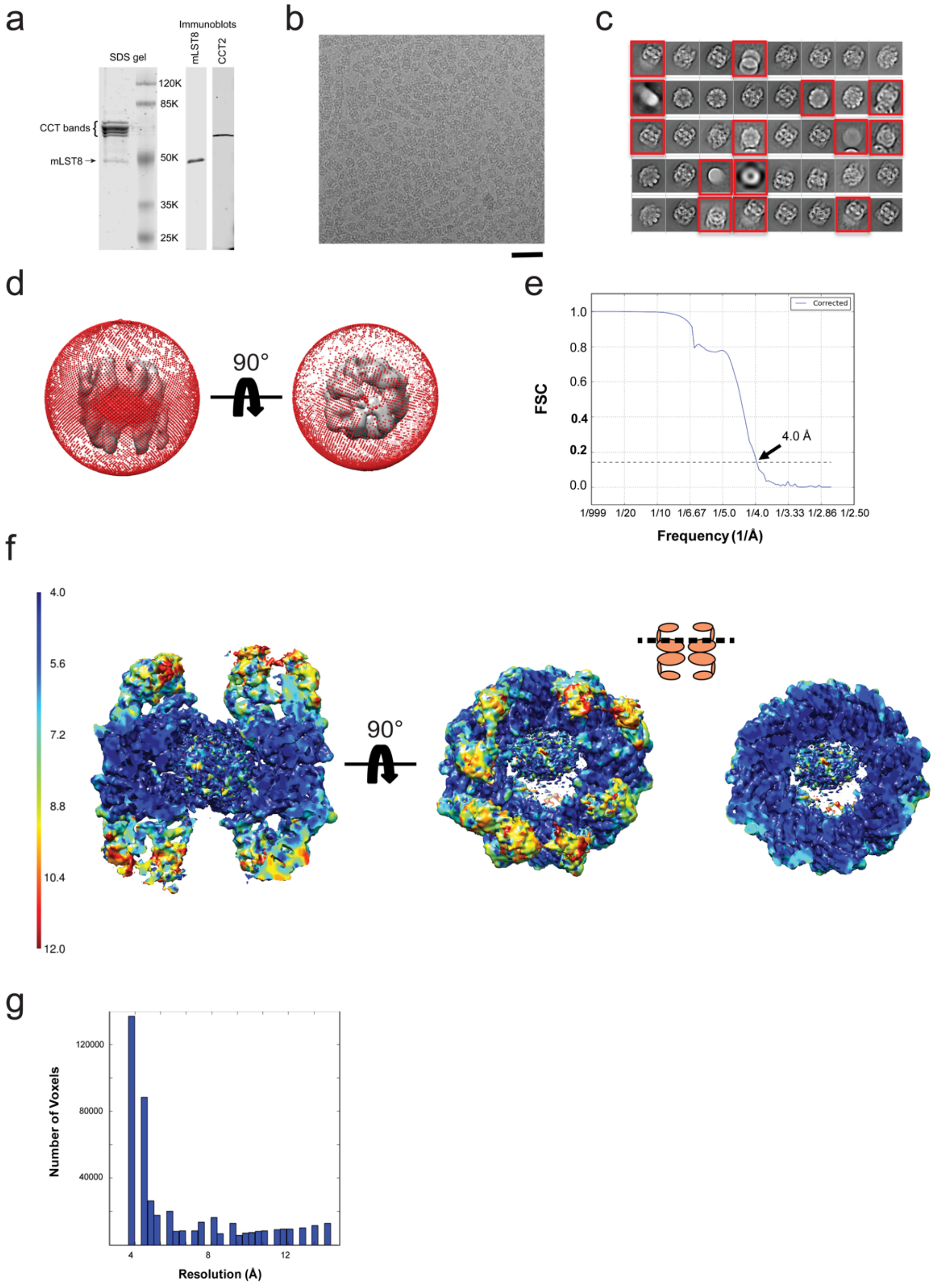

Supplementary Figure 4. Cryo-EM image analysis of the mLST8-CCT complex. (a) Electrophoretic analysis of the purified mLST8-CCT complex, showing an SDS gel (left) and immunoblots for mLST8 and CCT2. **(b)** An EM field of mLST8-CCT particles. Bar indicates 500 Å. **(c)** Maximum-likelihood 2D classification of the particles. Particles represented by the red-boxed classes were discarded from further processing. **(d)** Angular coverage of the particles used for the 3D reconstruction of the mLST8-CCT complex. **(e)** Plot of the FSC coefficients vs. Resolution between two independent reconstructions for the mLST8-CCT complex using the gold-standard method. The resolution obtained for a signal to noise ratio (SNR) of 0.143 (4.0 Å) is indicated. **(f)** Local resolution map of the mLST8-CCT complex. Three views of the mLST8-CCT complex color coded according to the level of resolution (left). Images correspond to the side (left), end on (center) and sliced end on (right) views. The position of the slice is indicated in the cartoon at the top. Images were obtained with MonoRes. **(g)** Histogram with the resolution reached for each voxel of the 3D reconstruction.

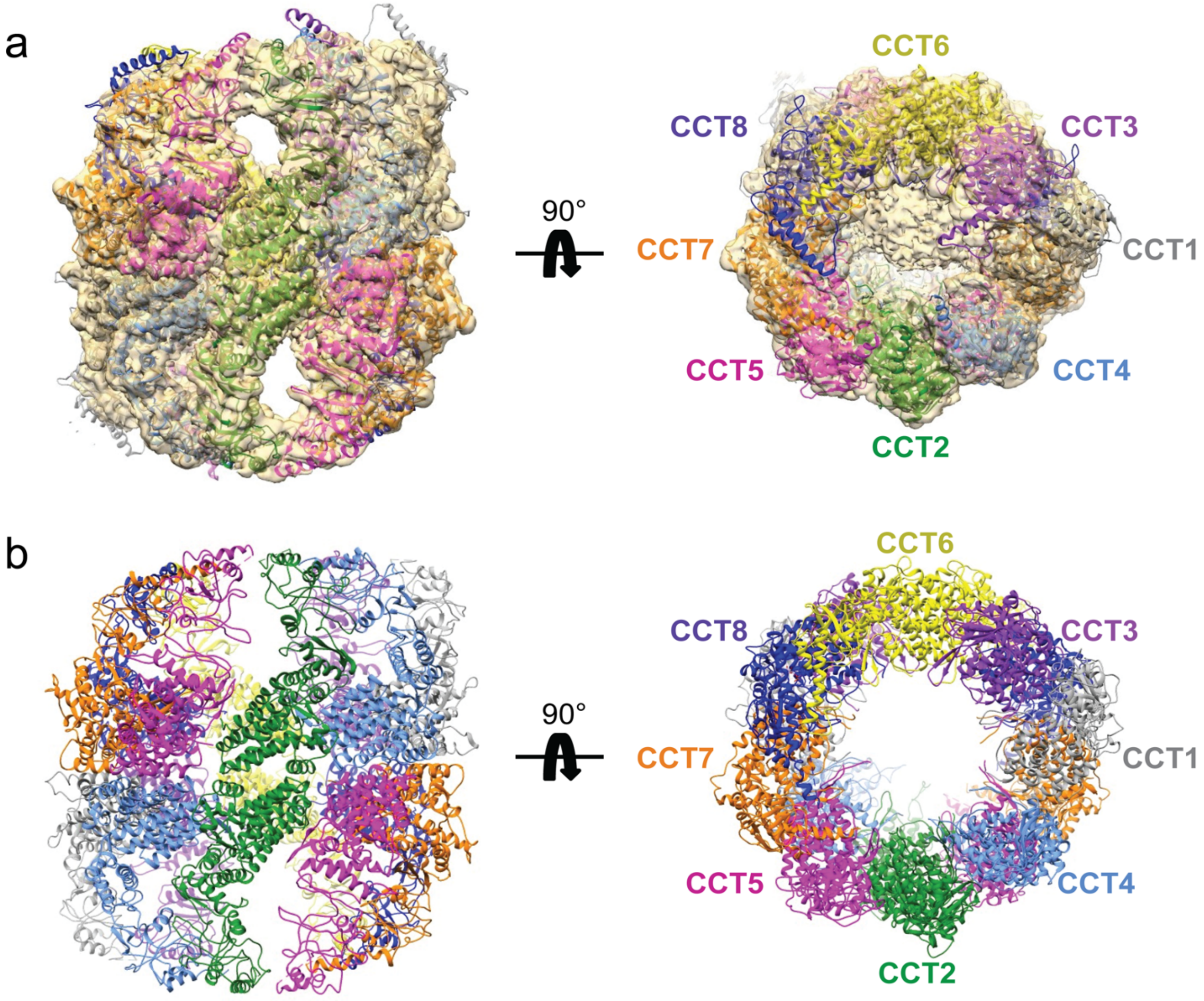

Supplementary Figure 5. Atomic model of the human CCT complex. (a) Docking of the atomic structure of yeast apo-CCT (PDB 5GW5) into the 3D reconstruction of the mLST8-CCT complex. **(b)** Atomic model of the human apo-CCT generated by homology-modelling from the yeast apo-CCT and subjected to flexible fitting and refinement.

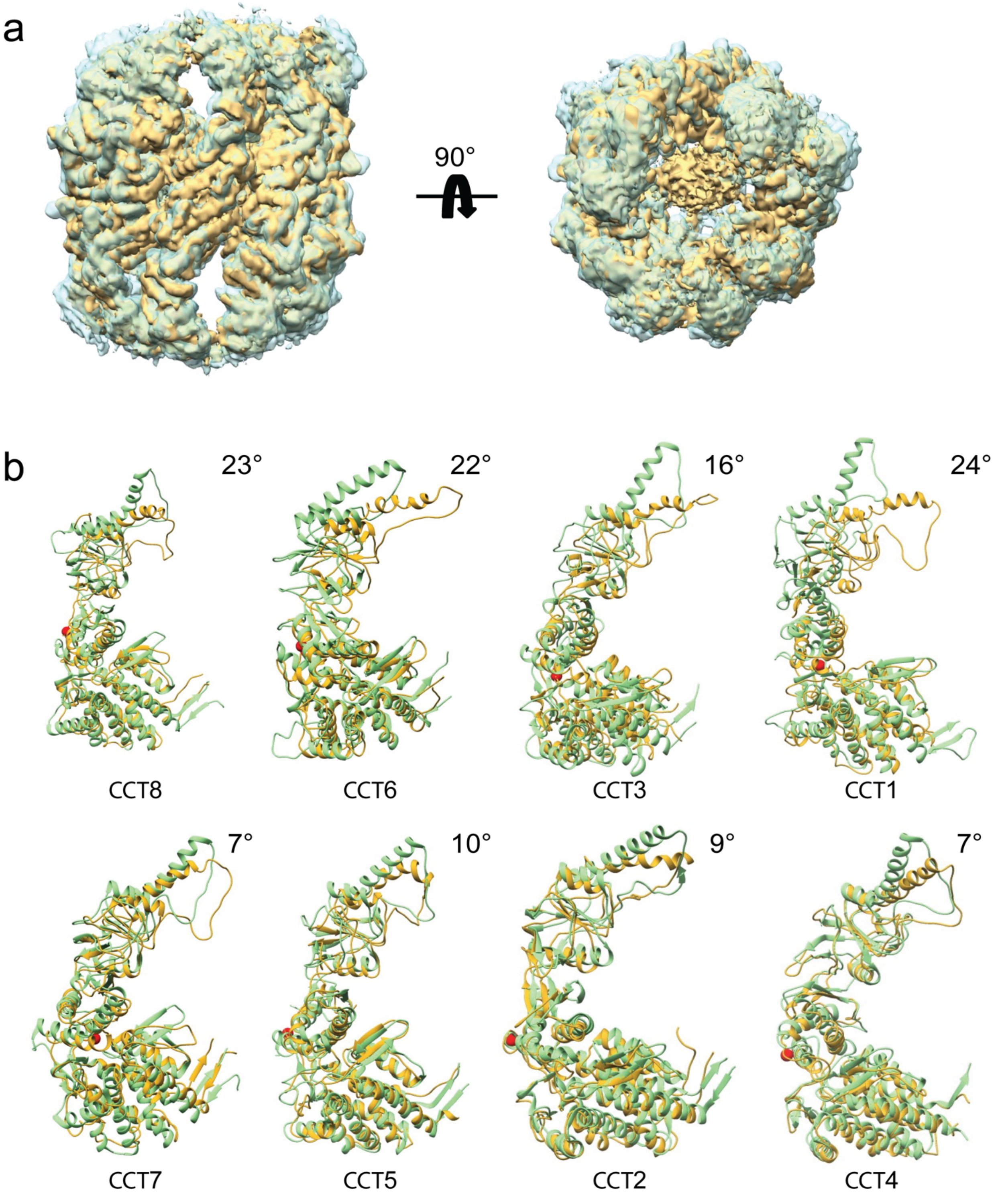

Supplementary Fig. 6. Comparison of yeast AMP-PNP CCT and human mLST8-CCT. (a) Superimposition of yeast AMP-PNP CCT (EMD 9541, green) and human mLST8-CCT (yellow) reconstructions. **(b)** Angular differences between the apical domains of the eight CCT subunits from the two structures (color coded as in panel a). Rotation angles were calculated with Chimera using the hinge colored in red as a reference axis.

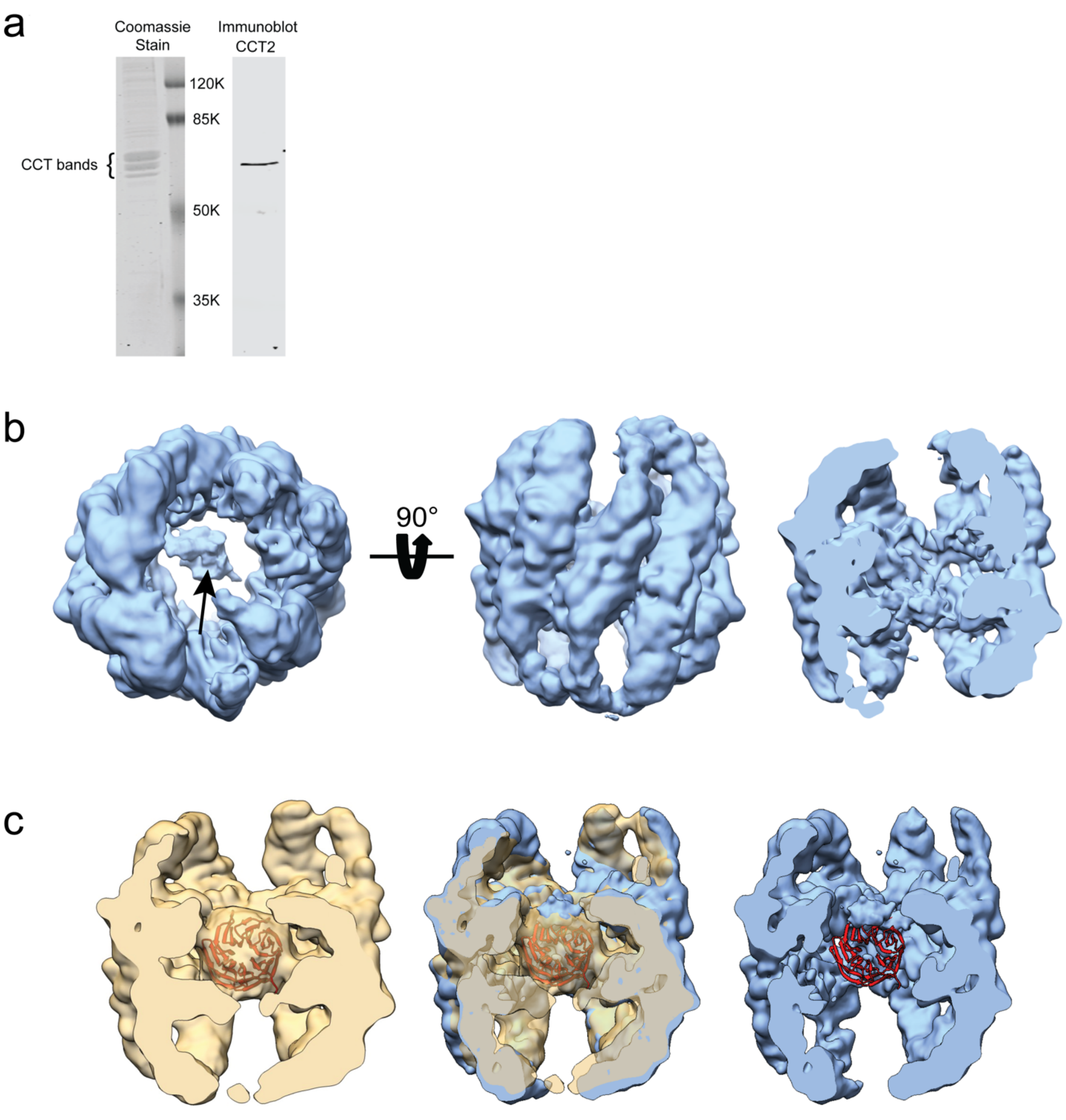

Supplementary Fig. 7. Comparison of substrate-free CCT and mLST8-CCT. (a) Electrophoretic analysis of the purified substrate-free CCT complex, showing an SDS gel (left) and an immunoblot for CCT2 (right). **(b)** Three different views of the 3D reconstruction of substrate-free CCT (7.5 Å resolution), showing end-on (left), side (center) and cut away side views (right). The arrow indicates a mass in the interior of the cavity much smaller than that of mLST8. **(c)** Left, cut away side view of the mLST8-CCT complex with the resolution limited to that of substrate free CCT. The atomic structure of mLST8 (PDB 4JT6) is docked into the mass located between the CCT rings. Center, an over-lay of mLST8-CCT and substrate-free CCT in the same cut-away view. Right, the same view of the substrate-free CCT structure with the mLST8 molecule positioned at the same place as in the mLST8-CCT structure, showing a lack of density attributable to mLST8 in the substrate-free structure.

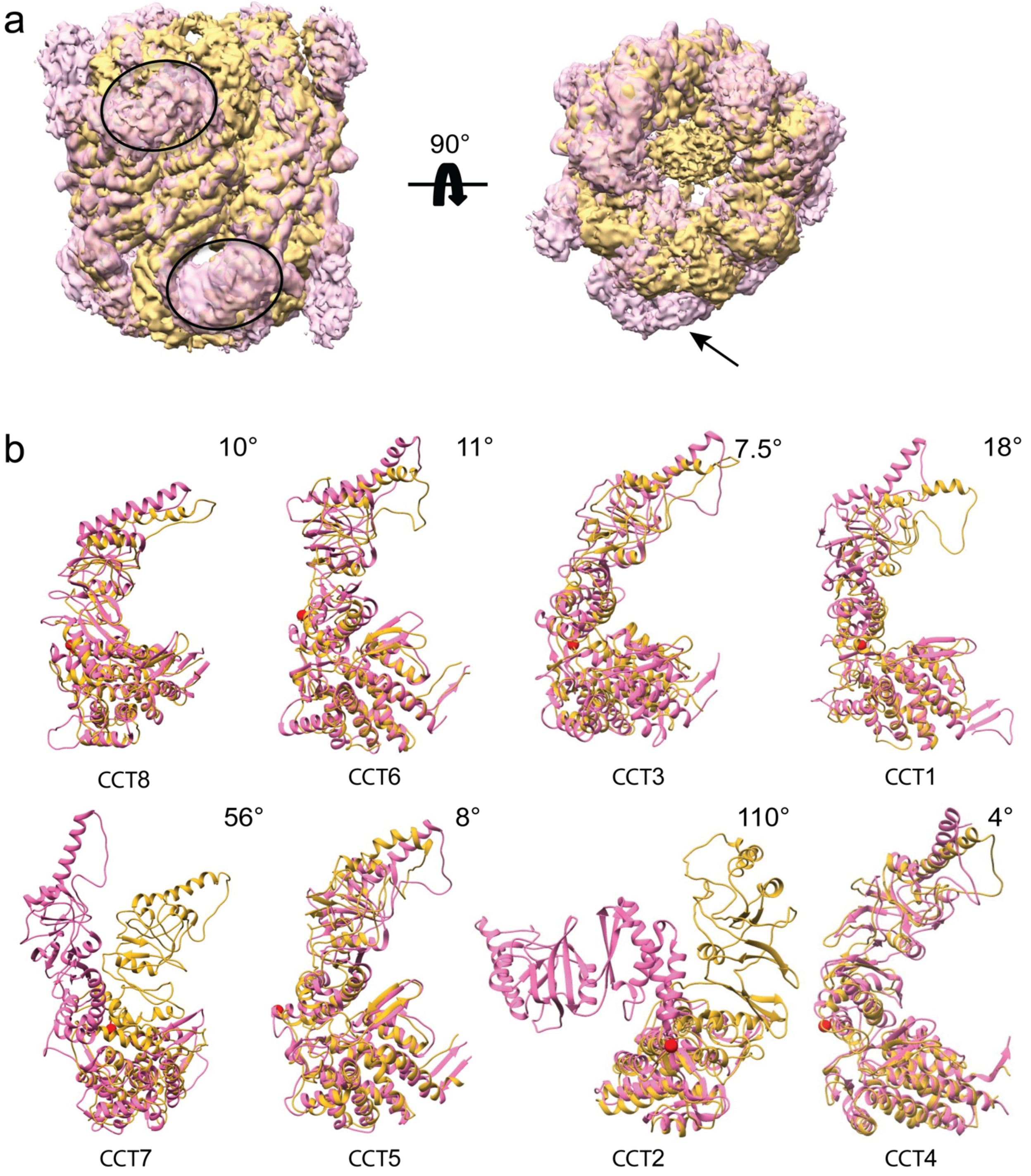

Supplementary Fig. 8. Comparison of yeast apo-CCT and human CCT-mLST8. (a) Superimposition of yeast NPP-CCT (EMD 9540, pink) and human mLST8-CCT (yellow) 3D reconstructions. The Z-shaped conformation of the CCT2 subunits in the yeast CCT is marked with circles (side view) or an arrow (end-on view). **(b)** Angular differences between the apical domains of the eight CCT subunits from the two structures (color coded as in panel a). Rotation angles were calculated with Chimera using the hinge colored in red as a reference axis.

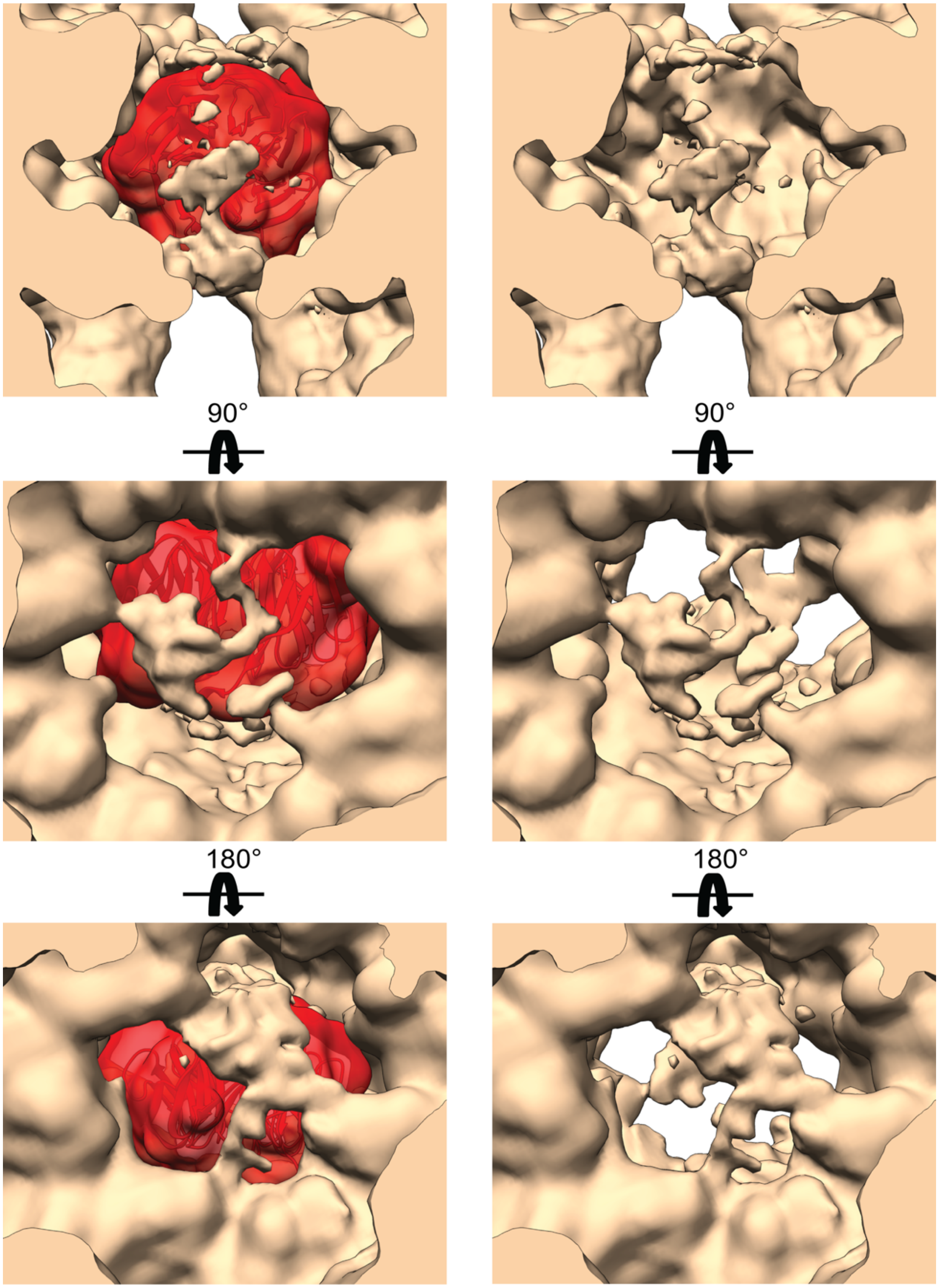

Supplementary Fig. 9. Location of the mLST8 molecule at the center of the CCT cavity. Left – The position of mLST8 (red) docked between the CCT rings (tan) is shown, filtered to the resolution of the mLST8-CCT complex, in a cut-away side view (top) and the two end-on views (middle and bottom). Right – Images are the same but with the mLST8 structure subtracted from the reconstruction, showing the small amount of remaining mass surrounding the mLST8 molecule.

Supplementary Table 1. Data collection and 3D reconstruction parameters

Data collection	CCT-mLST8	Apo-CCT
Microscope	Titan Krios	Titan Krios
Voltage (keV)	300	300
Nominal magnification	105,000x	130,000x
Detector	K2 Summit	K2 Summit
Pixel size (Å)	1.36	1.06
Defocus range (μm)	-1.5 to -3.0	-1.5 to -3.0
Electron dose (e^{-}/Å^{2})	36	42
Initial particles (no.)	1,769,600	504,060
Final particles (no.)	452,000	139,819
Global resolution (Å)	4.0	7.5
Local Resolution (Å)<br>Range	3.7-11.9	
Mean	5.57	
Median	4.6	

Supplementary Table 2. Model refinement and statistics

MODEL	CCT-mLST8	
**Composition**		
Chains	18	
Atoms	60249	
Residues	Protein: 7895 Nucleotide: 0	
Ligands	ADP:2	
**Bonds (RSMD)**		
Length (Å)	0.015	
Angles (º)	1.589	
MolProbity Score	2.54	
Clash score	10.97	
**Ramachandran plot (%)**		
Outliers	0.36	
Allowed	9.59	
Favored	90.05	
Rotamer outliers (%)	3.73	
Cß outliers (%)	0.14	
**Peptide plane (%)**		
Cis proline/general	0/0.1	
Twisted proline/general	1.0/0.4	
CaBLAM outliers (%)	4.9	
**ADP (B-factors)**		
Iso/Aniso	60249/0	
min/max/mean		
Protein	24.3/440.0/251.2	
Ligand	73.52/341.37/169.0	
**Occupancy**		
Mean	1.0	
Occ=1 (%)	100	
DATA		
**Box**		
Lengths (Å)	176.73, 191.8, 190.43	
Angles (º)	90.0, 90.0, 90.0	
Supplied Resolution (Å)	4.0	
Resolution estimates (Å)	Masked	Unmasked
d FSC (half maps; 0.143)	4.0	4.2
d 99 (full/half/half2)	4.3/8.9/8.9	4.2/9.2/9.2
d model	2.2	2.3
D FSC model (0/0.143/0.5)	2.2/3.0/4.1	2.2/3.0/4.2
Map min/max/mean	-0.67/2.53/0.04	
MODEL vs DATA		
CC (mask)	0.87	
CC (box)	0.9	
CC (peaks)	0.76	
CC (volume)	0.87	
Mean CC for ligands	0.87	

Supplementary Table 3. XL-MS crosslinks

Protein 1	Lysine Residue	Protein 2	Lysine Residue	Distance (Å)	Charge	Sequence of Highest scoring peptide	pLink Score	Mass error (Da)	Mass error (ppm)
**mLST8-CCT crosslinks**									
CCT1	532	mLST8	215	< 29.8	6	IDDLIKLHPESK(6)-TKIPAHTR(2)	4.55E-02	0.000539	0.217298
CCT1	538	mLST8	215	< 29.8	5	LHPESKDDK(6)-TKIPAHTR(2)	3.57E-03	-0.000953	-0.4476
CCT3	20	mLST8	215	30.7	4	KVQSGNINAAK(1)-TKIPAHTR(2)	2.82E-02	0.001497	0.683488
CCT4	21	mLST8	215	< 35.9	5	TKIPAHTR(2)-GKGAYQDR(2)	3.15E-01	0.000893	0.453963
CCT6	10	mLST8	215	< 42.0	5	TLNPKAEVAR(5)-TKIPAHTR(2)	3.24E-01	0.002596	1.202281
**mLST8 intralinks**									
mLST8	215	mLST8	245	14.6	4	FSPDSTLLATCSADQTCKIWR(18)-TKIPAHTR(2)	1.18E-08	0.019661	5.569954
mLST8	245	mLST8	305	16.9	4	FSPDSTLLATCSADQTCKIWR(18)-LWCVETGEIKR(10)	1.48E-03	0.004665	1.170661
mLST8	261	mLST8	305	17.5	4	TSNFSLMTELSIKSGNPGESSR(13)-LWCVETGEIKR(10)	1.66E-03	-0.019372	-4.990237
mLST8	215	mLST8	261	24.9	4	TSNFSLMTELSIKSGNPGESSR(13)-TKIPAHTR(2)	2.00E-09	0.002241	0.658588
mLST8	215	mLST8	305	31.1	5	LWCVETGEIKR(10)-TKIPAHTR(2)	1.39E-07	-0.006529	-2.65041
mLST8	86	mLST8	215	31.6	5	MYDLNSNNPNPIISYDGVNKNIASVGFHEDGR(20)-TKIPAHTR(2)	7.07E-06	0.015888	3.436474
mLST8	215	mLST8	313	34.6	6	EYGGHQKAVVCLAFNDSVLGGGEDQVDPR(7)-TKIPAHTR(2)	3.37E-04	0.003628	0.868343
mLST8	86	mLST8	305	37.3	5	MYDLNSNNPNPIISYDGVNKNIASVGFHEDGR(20)-LWCVETGEIKR(10)	4.25E-03	0.021766	4.285961
**CCT Equatorial Domain crosslinks**									
CCT1	515	CCT1	126	8.7	4	SLKFATEAAITILR(3)-LACKEAVR(4)	1.90E-09	0.005438	2.077575
CCT1	466	CCT1	494	9.6	4	SLLVIPNTLAVNAAQDSTDLVAKLR(23)-WIGLDLSNGKPR(10)	2.45E-02	0.027026	6.567223
CCT2	40	CCT2	50	10.3	4	LTSFIGAIAIGDLVKSTLGPK(15)-GMDKILLSSGR(4)	2.00E-07	0.026236	7.655668
CCT6	502	CCT6	127	10.4	5	KQLLHSCTVIATNILLVDEIMR(1)-IITEGFEAAKEK(10)	1.93E-03	0.020263	5.000424
CCT4	497	CCT4	55	10.5	4	KGGISNILEELVVQPLLVSVSALTLATETVR(1)-TSLGPKGMDK(6)	3.77E-05	0.014153	3.201701
CCT6	129	CCT6	426	10.6	5	EKALQFLEEVK(2)-HKPSVKGR(2)	3.11E-04	0.014272	5.998304
CCT6	424	CCT6	129	10.7	6	NAIDDGCVVPGAGAVEVAMAEALIKHKPSVK(25)-EKALQFLEEVK(2)	2.95E-09	0.016085	3.483539
CCT5	64	CCT2	522	10.8	5	MMVDKDGDVTVTNDGATILSMMDVDHQIAK(5)-VDNIIKAAPR(6)	9.82E-04	0.018384	4.088701
CCT8	490	CCT8	466	10.8	5	NVGLDIEAEVPAVKDMLEAGILDTYLGK(14)-ANEVISKLYAVHQEGNK(7)	4.60E-03	0.017852	3.562845
CCT6	502	CCT6	138	10.9	5	KQLLHSCTVIATNILLVDEIMR(1)-ALQFLEEVKVSR(9)	3.13E-05	0.033682	8.144915
CCT3	424	CCT3	127	11.3	5	NVLLDPQLVPGGGASEMAVAHALTEKSK(26)-KALDDMISTLK(1)	8.11E-06	0.043247	10.286559
CCT2	431	CCT2	119	11.5	3	TPGKEAVAMESYAK(4)-EAESLIAKK(8)	1.48E-17	0.005806	2.21651
CCT8	62	CCT7	77	11.5	6	MVINHLEKLFVTNDAATILR(8)-LLDVVHPAAKTLVDIAK(10)	4.90E-05	0.035406	8.329895
CCT2	431	CCT2	120	11.5	4	TPGKEAVAMESYAK(4)-KIHPQTIIAGWR(1)	1.12E-13	0.009521	3.120935
CCT8	421	CCT8	138	12.2	5	LVPGGGATEIELAKQITSYGETCPGLEQYAIK(14)-KAHEILPNLVCCSAK(1)	3.42E-01	0.074538	14.109687
CCT7	109	CCT7	440	12.7	5	QVKPYVEEGLHPQIIIR(3)-QQLLIGAYAKALEIIPR(10)	4.69E-06	0.023175	5.700544
CCT7	55	CCT7	47	13.3	4	GKATISNDGATILK(2)-GMDKLIVDGR(4)	8.34E-09	0.00581	2.20961
CCT3	506	CCT3	137	13.7	3	LQTYKTAVETAVLLLR(5)-ALDDMISTLKK(10)	5.99E-16	0.024719	7.717751
CCT6	129	CCT6	430	14.2	5	EKALQFLEEVK(2)-HKPSVKGR(6)	4.26E-07	0.001455	0.611514

CCT7	521	CCT7	77	14.6	5	INALTAASEAACLIVSVDETIKNPR(22)-LLDVVHPAAKTLVDIAK(10)	1.72E-07	0.017946	3.904248
CCT4	126	CCT5	42	15.9	5	LLQKGIHPTIISESFQK(4)-SHIMAAKAVANTMR(7)	2.45E-05	0.00088	0.245193
CCT4	497	CCT1	126	16.3	4	KGGISNILEELVVQPLLVSVSALTLATETVR(1)-LACKEAVR(4)	4.93E-02	0.047152	10.88096
CCT2	441	CCT2	119	16.3	3	EAVAMESYAKALR(10)-EAESLIAKK(8)	3.81E-09	0.011506	4.486883
CCT3	43	CCT6	430	16.4	5	TCLGPKSMMK(6)-HKPSVKGR(6)	4.56E-01	0.002584	1.175528
CCT4	143	CCT4	489	16.6	5	ALEKGIEILTDMSRPVELSDR(4)-HAQGEKTAGINVR(6)	6.60E-11	0.025127	6.439322
CCT1	33	CCT3	20	16.8	4	SQNVMAAASIANIVKSSLGPVGLDK(15)-KVQSGNINAAK(1)	7.41E-03	0.026584	7.090765
CCT7	440	CCT1	111	17.6	5	QQLLIGAYAKALEIIPR(10)-QKIHPTSVISGYR(2)	4.40E-05	-0.001158	-0.327852
CCT8	476	CCT8	138	17.8	6	LYAVHQEGNKNVGLDIEAEVPAVK(10)-KAHEILPNLVCCSAK(1)	2.04E-06	0.021018	4.689015
CCT5	529	CCT5	535	17.9	3	MILKIDDIR(4)-KPGESEE(1)	1.62E-04	0.009456	4.632734
CCT7	106	CCT1	111	18.4	5	SQDAEVGDGTTSVTLLAAEFLKQVK(22)-QKIHPTSVISGYR(2)	1.48E-02	0.007903	1.86822
CCT5	439	CCT5	150	18.5	5	VVYGGGAAEISCALAVSQEADKCPTLEQYAMR(22)-VAIEHLDKISDSVLVDIK(8)	1.74E-06	0.000605	0.108505
CCT8	466	CCT1	494	18.6	5	ANEVISKLYAVHQEGNK(7)-WIGLDLSNGKPR(10)	5.81E-05	0.014709	4.319987
CCT2	441	CCT2	120	18.7	4	EAVAMESYAKALR(10)-KIHPQTIIAGWR(1)	2.88E-04	0.044872	14.919072
CCT5	496	CCT2	135	19.4	4	EMNPALGIDCLHKGTNDMK(13)-EATKAAR(4)	9.87E-02	0.002926	0.962644
CCT4	55	CCT1	126	19.7	3	TSLGPKGMDK(6)-LACKEAVR(4)	2.65E-14	-0.000055	-0.025979
CCT2	119	CCT2	119	19.8	3	EAESLIAKK(8)-EAESLIAKK(8)	9.79E-02	0.011129	5.234047
CCT8	459	CCT3	20	20.3	4	ALAENSGVKANEVISK(9)-KVQSGNINAAK(1)	1.59E-02	-0.004585	-1.576333
CCT4	139	CCT5	483	20.8	5	GIHPTIISESFQKALEK(13)-QVKEMNPALGIDCLHK(3)	7.83E-06	0.028625	7.339527
CCT5	35	CCT1	111	20.9	5	LMGLEALKSHIMAAK(8)-QKIHPTSVISGYR(2)	5.37E-04	0.02193	6.777368
CCT4	143	CCT5	483	21.2	6	ALEKGIEILTDMSRPVELSDR(4)-QVKEMNPALGIDCLHK(3)	1.32E-07	0.029521	6.767379
CCT2	120	CCT2	522	21.9	5	KIHPQTIIAGWR(1)-VDNIIKAAPR(6)	1.68E-10	0.006754	2.533759
CCT1	466	CCT8	466	22.3	5	SLLVIPNTLAVNAAQDSTDLVAKLR(23)-ANEVISKLYAVHQEGNK(7)	5.32E-05	0.026221	5.627385
CCT6	449	CCT6	426	24.8	5	AQLGVQAFADALLIIPKVLAQNSGFDLQETLVK(17)-HKPSVK(2)	1.56E-05	0.019209	4.410273
CCT2	120	CCT2	135	25.0	3	KIHPQTIIAGWR(1)-EATKAAR(4)	7.90E-01	-0.013529	-5.676667
CCT1	111	CCT1	126	25.6	5	QKIHPTSVISGYR(2)-LACKEAVR(4)	2.00E-05	0.006848	2.665222
CCT2	40	CCT2	119	26.2	4	LTSFIGAIAIGDLVKSTLGPK(15)-EAESLIAKK(8)	8.79E-13	0.040814	12.601075
CCT3	47	CCT3	506	27.9	5	TCLGPKSMMKMLLDPMGGIVMTNDGNAILR(10)-LQTYKTAVETAVLLLR(5)	5.36E-01	0.05183	9.748527
CCT2	40	CCT2	120	28.0	5	LTSFIGAIAIGDLVKSTLGPK(15)-KIHPQTIIAGWR(1)	6.97E-06	0.032506	8.856775

Supplementary Table 4. sgRNA, siRNA and qPCR information

sgRNA Target	Sequence
**Non-targeting**	GGTCTTCGAGAAGACCT
**CCT5**	TGGAGATGGAACCACAGGAG
**PhLP1**	CACCCTTGATGATAAGTTGC
**PhLP1**	AGAAGCTGTCAATGACTTGC
**CCT3**	CCTGCACCATTCTCCTCCGG
**Raptor**	CCACGTCTGCAGGTCGTATA
**Raptor**	CCCGTTCCTCCTTGGCGTTC
**Raptor**	GTCTACGACTGTTCCAATGC

siRNA Target	Vendor	Product Number
**Non-Targeting**	Thermo Fisher	AM4635
**mLST8**	Qiagen	SI00425474
**mLST8**	Qiagen	SI04213923
**Raptor**	Dharmacon	L-004107-00-0005
**CCT1**	Dharmacon	L-012749-00-0005
**CCT5**	Dharmacon	L-012797-00-0005

qPCR PrimeTime Assay (IDT)	Product Number
**HPRT1**	196118969
**RPTOR**	196118965
**MTOR**	201378535
**MLST8**	196118974

Supplementary Table 5. Antibody information

Antibody Target	Vendor	Product Number	Dilution
**AKT**	Cell Signaling	9272	1:500 WB
**AKT P-Ser473**	Cell Signaling	4060	1:500 WB
**c-Myc**	Invitrogen	13-2500	1:1000 WB
**CCT2**	Abcam	Ab92746	1:10000 WB
**CCT4**	Abcam	Ab129072	1:5000 WB
**CCT5**	Abcam	Ab129016	1:10000 WB
**CCT5**	BioRAD	MCA2178	1:100 IP
**Flag**	Sigma	F3165	1:2000 WB 1:100 IP
**GAPDH**	BioRAD	MCA4740	1:2000 WB
**GFP**	Abcam	Ab6556	1:5000 WB
**HA**	Roche	11867423001	1:500 WB 1:50 IP
**His**	Thermo Fisher	MA1-21315	1:1000 WB
**IRS-1**	Cell Signaling	2390	1:500 WB
**IRS-1 P-**	Cell Signaling	2388	1:500 WB
**mLST8**	Cell Signaling	3274	1:500 WB
**mSin1**	Cell Signaling	D7G1A	1:500 WB
**mTOR**	Cell Signaling	2972	1:500 WB
**PhLP1 (N-term)**	BMW Lab		1:2000 WB
**Raptor**	Cell Signaling	2280	1:500 WB
**Rictor**	Cell Signaling	2150	1:500 WB
**Strep**	Genscript	A01732	1:5000 WB
**V5**	Invitrogen	R960-25	1:2000 WB 1:100 IP
**IRDye 680RD Goat Anti-Rabbit**	LI-COR	926-68071	1:5000
**IRDye 800CW Goat Anti-Rabbit**	LI-COR	926-32211	1:5000
**IRDye 680RD Goat Anti-Mouse**	LI-COR	926-68070	1:5000
**IRDye 800CW Goat Anti-Mouse**	LI-COR	926-32210	1:5000
**IRDye 800CW Goat Anti-Rat**	LI-COR	926-32219	1:5000
**IRDye 800CW Donkey Anti-Goat**	LI-COR	926-32215	1:5000
