# Supplementary material for: Structural and functional analysis of the role of the chaperonin CCT in mTOR complex assembly
Source: Nat Commun. 2019 Jun 28;10:2865. doi: 10.1038/s41467-019-10781-1 (PMC6599039; doi:10.1038/s41467-019-10781-1)
Supplement: Supplementary file 3 — Description of Additional Supplementary Files [file 41467_2019_10781_MOESM3_ESM.pdf]

Description of Additional Supplementary Files

File name: Supplementary Movie 1

Description: Atomic structure of the human mLST8-CCT complex. The movie gives a three-dimensional view of the complex, displaying the position of mLST8 between the CCT rings.
