# Supplementary material for: Structural and functional analysis of the role of the chaperonin CCT in mTOR complex assembly
Source: Nat Commun. 2019 Jun 28;10:2865. doi: 10.1038/s41467-019-10781-1 (PMC6599039; doi:10.1038/s41467-019-10781-1)
Supplement: Supplementary file 5 — Reporting Summary [file 41467_2019_10781_MOESM5_ESM.pdf]

natureresearch

Corresponding author(s): Barry M. Willardson and Jose M. Valpuesta

Last updated by author(s): May 9, 2019

Reporting Summary

Nature Research wishes to improve the reproducibility of the work that we publish. This form provides structure for consistency and transparency in reporting. For further information on Nature Research policies, see Authors & Referees and the Editorial Policy Checklist.

Statistics

For all statistical analyses, confirm that the following items are present in the figure legend, table legend, main text, or Methods section.

n/a	Confirmed	
☐	☒	The exact sample size (n) for each experimental group/condition, given as a discrete number and unit of measurement
☐	☒	A statement on whether measurements were taken from distinct samples or whether the same sample was measured repeatedly
☐	☒	The statistical test(s) used AND whether they are one- or two-sided<br>*Only common tests should be described solely by name; describe more complex techniques in the Methods section.*
☒	☐	A description of all covariates tested
☒	☐	A description of any assumptions or corrections, such as tests of normality and adjustment for multiple comparisons
☐	☒	A full description of the statistical parameters including central tendency (e.g. means) or other basic estimates (e.g. regression coefficient) AND variation (e.g. standard deviation) or associated estimates of uncertainty (e.g. confidence intervals)
☐	☒	For null hypothesis testing, the test statistic (e.g. F, t, r) with confidence intervals, effect sizes, degrees of freedom and P value noted<br>*Give P values as exact values whenever suitable.*
☒	☐	For Bayesian analysis, information on the choice of priors and Markov chain Monte Carlo settings
☒	☐	For hierarchical and complex designs, identification of the appropriate level for tests and full reporting of outcomes
☒	☐	Estimates of effect sizes (e.g. Cohen's d, Pearson's r), indicating how they were calculated

Our web collection on statistics for biologists contains articles on many of the points above.

Software and code

Policy information about availability of computer code

Data collection	No software was used in the data collection.
Data analysis	Immunoblot intensities were quantified using the LICOR Image Studio software. Real-time PCR results were analyzed using the QuantStudio 5 analysis software. These programs are part of instrument software.<br>The XL-MS spectra were analyzed using the pLink 2 software. The movies of mLST8-CCT complex were aligned using MotionCorr24 program as part of the Scipion processing workflow. The MotionCorr2 output was subjected to CTF correction using CTFFIND4. Particles were automatically picked with Xmipp. A first 2D classification using Relion 2.0 and some of the best 2D classes were used as a template to generate an initial model using both EMAN and RANSAC. Local resolution in the 3D structure of the mLST8-CCT complex was estimated using MonoRes from Xmipp package and ResMap.<br>Models for each human CCT chain were generated using SWISS-MODEL homology-modelling server using the yeast CCT structure (PDB 5GW5) as a reference. The resulting model was docked into the cryo-EM density using Chimera and further subjected to flexible fitting of the individual subunits with iMODFIT. Manual adjustment and real-space refinement were carried out in COOT to increase the quality of the fitting. The resulting model was refined by several rounds using PHENIX and CCP-EM software suites. Validation of the final model was done using the phenix.validation_cryoem module in PHENIX. The NCBI Blastp web-based suite was used to perform an alignment of the eight human CCT subunits. All of these programs have been described in the published literature and are referenced in the methods.

For manuscripts utilizing custom algorithms or software that are central to the research but not yet described in published literature, software must be made available to editors/reviewers. We strongly encourage code deposition in a community repository (e.g. GitHub). See the Nature Research guidelines for submitting code & software for further information.

Data

Policy information about availability of data

All manuscripts must include a data availability statement. This statement should provide the following information, where applicable:

- Accession codes, unique identifiers, or web links for publicly available datasets
- A list of figures that have associated raw data
- A description of any restrictions on data availability

Cryo-EM data have been deposited in the Electron Microscopy Data Bank under accession codes EMD-4489 and EMD-4503 for the human mLST8-CCT complex and human apo-CCT, respectively. The associated atomic model for CCT-mLST8 has been deposited in the Protein Data Bank under accession code PDB 6QB8. The mass spectrometry data has been deposited in the public data repository chorusproject.org Project ID: 1567 Name: mLST8-CCT Experiment ID: 3379 Name: TA_DSS_MC_XL. Other data will be made available to qualified individuals making reasonable requests.

Field-specific reporting

Please select the one below that is the best fit for your research. If you are not sure, read the appropriate sections before making your selection.

☒ Life sciences ☐ Behavioural & social sciences ☐ Ecological, evolutionary & environmental sciences

For a reference copy of the document with all sections, see nature.com/documents/nr-reporting-summary-flat.pdf

Life sciences study design

All studies must disclose on these points even when the disclosure is negative.

Sample size	For immunoblotting, immunoprecipitation and qPCR, the sample size was not pre-calculated, but was determined empirically by the number of replicates required for the data to converge.
Data exclusions	Failed immunoblots that could not be quantified because of low signal to noise ratios were excluded. Of the blots that could be quantified, statistical outliers were identified and excluded using the Grubbs test, which resulted in exclusion of only 2 data points among 746 reported values.
Replication	For immunoblots, immunoprecipitation, and qPCR, distinct replicates were done until the data converged.
Randomization	For immunoblots, immunoprecipitation, and qPCR, wells of HEK-293T cells were randomly selected for CCT or PhLP1 depletion and transfection with mTOR subunits. Randomization was not applicable for the structural studies.
Blinding	Blinding was not practical for the biochemical experiments in which one investigator was responsible to perform experiments and analyze the data. However, the blots were regularly checked for accuracy by a second investigator. Blinding was not applicable for the structural studies.

Reporting for specific materials, systems and methods

We require information from authors about some types of materials, experimental systems and methods used in many studies. Here, indicate whether each material, system or method listed is relevant to your study. If you are not sure if a list item applies to your research, read the appropriate section before selecting a response.

Materials & experimental systems

n/a	Involved in the study	
☐	☒	Antibodies
☐	☒	Eukaryotic cell lines
☒	☐	Palaeontology
☒	☐	Animals and other organisms
☒	☐	Human research participants
☒	☐	Clinical data

Methods

n/a	Involved in the study	
☒	☐	ChIP-seq
☒	☐	Flow cytometry
☒	☐	MRI-based neuroimaging

Antibodies

Antibodies used	Antibodies are described as follows: name, host, supplier product number. 1) Akt, rabbit, Cell Signaling Techology, 9272. 2) Akt phospho-Ser473, rabbit, Cell Signaling Technology, 4060. 3) c-Myc-tag, mouse, Invitrogen 13-2500. 4) CCT2, rabbit, Abcam, ab92746. 5) CCT5, rabbit, Abcam, ab129016. 6) CCT5, rat, AbD Serotec, MCA2178. 7) FLAG, mouse, Sigma, F3165. 8) GAPDH, mouse, AbD Serotec, MCA4740. 9) GFP, rabbit, Abcam, ab6556. 10) HA-tag, rat, Roche, 11867423001. 11) His-tag, mouse, Thermo, MA1-21315. 12) IRS1, rabbit, Cell Signaling Technology, 2382. 13) IRS1 Phospho-Ser636/639, rabbit, Cell Signaling Technology, 2388. 14) mLST8, rabbit, Cell Signaling Technology, 3274. 15) mTOR, rabbit, Cell Signaling Technology, 2972. 16) PhLP1 N-term. 1-50, made in house, described in Thulin et al. (1999) Mol. Vis. 5:40. 17) Raptor, rabbit, Cell Signaling

	Technology, 2280. 18) Rictor, rabbit, Cell Signaling Technology, 2140. 19) Strep-tag, mouse, Genscript, A01732. 20) V5-tag, mouse, Invitrogen, R960-25.
Validation	Antibodies were all validated by the manufacturer to recognize the human target protein. They readily recognized the appropriately sized band in our lab with no non-specific bands in the same region of the blot. Antibodies to epitope tags were validated by the manufacturer and in our lab to specifically recognize the appropriately sized band with no non-specific bands in the same region of the blot.

Eukaryotic cell lines

Policy information about cell lines

Cell line source(s)	HEK-293T and HepG2 cells were purchased from ATCC.
Authentication	Cells were authenticated by ATCC and carefully labeled and tracked in our lab. As stated in the methods, they were not used beyond 15 passages.
Mycoplasma contamination	Cells were not tested for mycoplasma contamination.
Commonly misidentified lines (See ICLAC register)	None
